# Neuronal mechanisms of epileptogenesis

**DOI:** 10.3389/fncel.2014.00029

**Published:** 2014-02-21

**Authors:** Roberto Di Maio

**Affiliations:** Department of Neurology, Pittsburgh Institute for Neurodegenerative Disease, Ri.MED Foundation, University of PittsburghPittsburgh, PA, USA

**Keywords:** epileptogenesis, neuronal epileptic damage, hippocampal damage, epilepsy, Temporal Lobe Epilepsy (TLE), TLE prevention

The primary purpose of this topic is to collect scientific contributions providing novel insights in the cellular and molecular mechanisms of epileptogenesis as potential targets for innovative therapeutic approaches aimed at preventing the chronic epileptic disorder.

Prevention of chronic epileptic disorder with an appropriate intervention might represent the most ambitious goal in the clinical treatment of this epileptic disorder, but has been largely unsuccessful to this point. Clinical trials aimed at prevention of chronic epilepsy have often produced negative, disappointing results. However, in most cases, these studies ultimately evaluated the downstream clinical manifestations, failing to monitor early, specific molecular epileptogenic events. Therefore, elucidation of the underlying mechanisms of epileptogenesis, are essential.

Several types of brain injuries are causes of acquired epilepsy, including brain trauma, one of the most common causes of idiopathic epilepsy (Hunt et al., 2013; Timofeev et al., 2013). Genetic mutations enhancing structural and functional alterations of key proteins including pre-synaptic complexes (Toader et al., 2013) and potassium channels (D'Adamo et al., 2013) are also related to the occurrence of epileptic disorders. Consistently with these findings obtained in genetic animal models of epilepsy, studies conducted in animal models of acquired epilepsy addressed the critical role of vesicular neurotransmitters transporters (VNTs) (Van Liefferinge et al., 2013) and non-neuronal potassium channel (Kir4.1) (Nagao et al., 2013) expression during epileptogenesis.

Temporal Lobe Epilepsy (TLE) is the most common form of refractory epileptic disorder often related to childhood seizures. The symptomatic manifestations of TLE appear only after a widespread irreversible damage of entorhinal cortex (Bartolomei et al., 2005), hippocampus (Mathern et al., 2002) and perirhinal cortex, which has a major role in the spread of limbic seizures (Biagini et al., 2013), These pathological features of TLE reduce the possibility of successful therapeutic approaches, often rendering the disease refractory. The difficult clinical management of chronic TLE and the limited success rate of surgical approaches, increase the incapacitating nature of this specific epileptic disorder.

Despite its complex etiology, a common feature of the epileptic disorders is a paroxysmal excitatory activity, which is able to produce the same pathological features that are ultimately recognized clinically as epileptic disease.

Only recently the role of oxidative stress in epilepsies has begun to be recognized. Neuronal hyper-excitability is associated with a calcium-dependent activation of intracellular oxidant systems, including NOX2, which is the major NMDAR-regulated source of superoxide (Di Maio et al., 2011). This early phenomenon occurring during the epileptic onset might be responsible for the long-term neuronal dysfunction leading to the chronic epileptic disorder (Di Maio et al., 2012).

Excitatory/inhibitory unbalance and oxidative-related events might be determinant in the epileptic pathogenesis of neuronal networks mediating a complex disruption of self-regulatory homeostatic mechanisms such as the bioenergetics systems (Boison et al., 2013).

Epileptic neurons may develop short and long-term adaptive changes in sensitivity to GABA-ergic neurotransmission by means of GABA_A_ receptor (Cifelli et al., 2013), worsening the excitatory/inhibitory unbalance and reducing the possibility of successful therapeutic approaches with the conventional Antiepileptic Drugs. Interesting insights have been recently provided on this regard. Epileptogenic changes of GABA_A_ receptor may be caused by altered expression of scaffolding proteins involved in the trafficking and anchoring of GABA_A_ receptors. This phenomenon could directly impact the stability of GABA-ergic synapses and promote impairment of the neuronal response to the inhibitory GABA-ergic input. These findings offer novel potential therapeutic targets to prevent the development of epilepsy.

Dopaminergic projections to limbic system play also a critical role in the control of seizures. Dopaminergic activity in limbic structure exerts a complex neuromodulation of neuronal excitability mainly through D1 and D2 receptors subtypes. Impairment of the fine tuning mediated by dopamine (DA) receptors activity can contribute to spread of seizures in the limbic system. Recent evidences on the identification of intracellular signaling pathways activated by DA receptors activity are leading to promising studies aimed at the identification of novel targets for the treatment of epilepsy (Bozzi and Borrelli, 2013).

An increasing number of experimental evidences suggest a major involvement of inflammation in epileptogenesis. Seizure activity elicits release of pro-inflammatory cytokines and activates immune responses. These phenomena have been widely related to an increased brain susceptibility to seizure, synaptic reorganization and neuronal death (Xu et al., 2013).

Inflammatory processes in brain can affect the extracellular neuronal matrix (ECM) integrity. ECM plays a critical role in the modulation of AMPA receptor mobility, paired-pulse depression, L-type voltage-dependent Ca^2+^ channel activity and LTP processes. Noteworthy, an original study published in this topic, suggests that changes in the expression of Hyaluronic acid, the major component of neuronal ECM, can lead to neuronal hyper-excitability and calcium dysregulation (Vedunova et al., 2013).

Neuronal cell death has been implicated as a causal factor leading to the development of the epileptic disorder. The findings reported in this topic support the idea that repeated seizures mediate neuronal necrosis and apoptosis prevalently associated to the activation of certain distinct anti/pro-apoptotic Bcl-2 family factors. Thus, epileptogenesis elicits apoptotic events by means of a specific pattern of Bcl-2 family proteins, which might represent a possible target of intervention to protect against the epileptic damage (Henshall and Engel, 2013).

Hormones play an important role in the epileptic disorders. Corticosteroids, progesterone, estrogens, and neurosteroids have been shown to affect seizure activity in animal models and in human. However, the impact of hormones on epileptogenesis is still underexplored and controversial. Further studies are required in the field to generate evidences on the therapeutic potential of hormonal agents in epileptogenesis (Reddy, 2013).

The circadian pattern of seizures is one of the first phenomena described in the epileptic disorders. However, due to the lack of promising hypotheses, has not attracted enough scientific attention. Recent findings provide novel insights in the implication of circadian rhythm in modulating transcription factors governing clock genes expression, and the mTOR signaling pathway, one of the most relevant signaling pathway in epilepsy (Cho, 2012).
